# A Spontaneous H2-Aa Point Mutation Impairs MHC II Synthesis and CD4^+^ T-Cell Development in Mice

**DOI:** 10.3389/fimmu.2022.810824

**Published:** 2022-03-04

**Authors:** Yun Zhao, Juan Xiong, Hai-Xia Chen, Min Zhang, Li-Na Zhou, Yin-Fang Wu, Wei-Jie Li, Xia Fei, Fei Li, Chen Zhu, Wen Li, Song-Min Ying, Lie Wang, Zhi-Hua Chen, Hua-Hao Shen

**Affiliations:** ^1^ Key Laboratory of Respiratory Disease of Zhejiang Province, Department of Respiratory and Critical Care Medicine, Second Affiliated Hospital of Zhejiang University School of Medicine, Hangzhou, China; ^2^ Department of Drug Candidate, Qihan Bio Inc., Hangzhou, China; ^3^ Institute of Immunology, Zhejiang University School of Medicine, Hangzhou, China; ^4^ International Institutes of Medicine, the Fourth Affiliated Hospital of Zhejiang University School of Medicine, Yiwu, China; ^5^ State Key Lab of Respiratory Disease, National Clinical Research Center for Respiratory Disease, Guangzhou, China

**Keywords:** MHC II, CD4^+^ T cells, immunodeficiency, H2-Aa, point mutation

## Abstract

Major histocompatibility complex class II (MHC II) is an essential immune regulatory molecule that plays an important role in antigen presentation and T-cell development. Abnormal MHC II expression can lead to immunodeficiency, clinically termed as type II bare lymphocyte syndrome (BLS), which usually results from mutations in the MHC II transactivator (CIITA) and other coactivators. Here, we present a new paradigm for MHC II deficiency in mice that involves a spontaneous point mutation on H2-Aa. A significantly reduced population of CD4^+^ T cells was observed in mice obtained from the long-term homozygous breeding of *autophagy-related gene microtubule-associated protein 1 light chain 3 β* (Map1*lc3b*, *Lc3b*) knockout mice; this phenotype was not attributed to the original knocked-out gene. MHC II expression was generally reduced, together with a marked deficiency of H2-Aa in the immune cells of these mice. Using cDNA and DNA sequencing, a spontaneous H2-Aa point mutation that led to false pre-mRNA splicing, deletion of eight bases in the mRNA, and protein frameshift was identified in these mice. These findings led to the discovery of a new type of spontaneous MHC II deficiency and provided a new paradigm to explain type II BLS in mice.

## Introduction

As an important immune regulator, major histocompatibility complex class II (MHC II), which is a heterodimer consisting of an α chain and a β chain, plays a key role in the immune response (1). MHC II genes are constitutively expressed in immune cells such as B cells, dendritic cells, and thymus epithelial cells (2). The expression of MHC II in B and dendritic cells is essential for antigen presentation (3), while MHC II expression in thymus epithelial cells contributes to CD4^+^ T-cell development (4). In addition, macrophages can express a higher level of MHC II following stimulation with interferon-γ (IFN-γ) (5). The aberrant expression of class II proteins has been implicated in immune dysfunction, and a lack of MHC II causes an immunodeficiency called type II BLS (6). CD4^+^ T cells are an important T-cell subtype. Hematopoietic stem cells differentiate into common lymphoid progenitor cells and migrate to the thymus which is the main site for lymphocyte development. Upon undergoing T-cell receptor (TCR) rearrangement, positive selection, and negative selection, mature T cells are generated and finally delivered to peripheral immune organs to execute immune function (7). MHC II plays an important role in T-cell selection, and only T cells with moderate affinity to MHC II but low affinity to MHC II self-antigen can ultimately develop into mature CD4^+^ T cells (8). Therefore, patients with type II BLS exhibit severely hampered T-cell activation and greatly reduced CD4^+^ T-cell counts.

MHC II transactivator (CIITA) is a non-DNA-binding protein factor that is recruited to the enhancer complex of MHC II genes and predominantly regulates MHC II expression. CIITA expression acts as a master switch in immune responses (9). CIITA interacts with DNA-binding proteins, such as RFX factors, including RFXANK/RFXB, RFX5, RFXAP, and CREB, to form an anchor in the large transcriptosome complex. Therefore, the abnormal expression of these proteins can result in MHC II deficiency, namely, type II BLS which has a very low incidence clinically. In previous reports, patients displayed some genetic defects in factors such as CIITA, RFXANK, RFX5, and RFXAP; nearly every patient had a specific defect in the transcription factor that is essential for MHC II expression. However, some atypical cases still exist wherein the genetic defect is undefined and the mutations in MHC II genes are not clear (10).

In the present study, we observed an immunodeficient phenotype in mice that were obtained from the homozygous breeding of *lc3b* knockout mice. The obtained mice showed impaired MHC II synthesis and diminished CD4^+^ T-cell development, which is unlikely to be due to impairment of the original gene. We also confirmed that the immunodeficiency in this mouse strain was not related to the CIITA transcriptosome complex, but resulted from a spontaneous *H2-Aa* point mutation, which led to the errant splicing of pre-mRNA and frameshift of protein.

## Results

**Figure 1:**
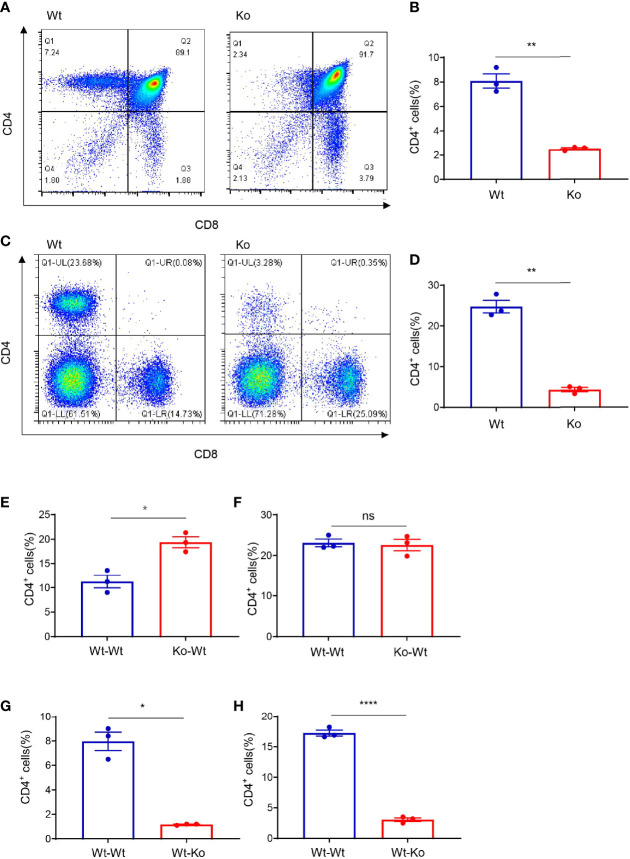
Thymus-dependent CD4^+^ T-cell deficiency was accidentally observed during long-term homozygous breeding of *lc3b* knockout mouse strain. **(A, C)** Representative images of flow cytometry of the thymus **(A)** and spleen **(C)** of wild-type and knockout mice. **(B, D)** Proportion of CD4^+^ T cells in the thymus **(B)** and spleen **(D)** of wild-type and knockout mice. **(E, F)** Proportion of CD4^+^ T cells in the thymus **(E)** and spleen **(F)** of wild-type mice (respectively receiving bone marrow from wild-type mice and knockout mice). **(G, H)** Proportion of CD4^+^ T cells in the thymus **(G)** and spleen **(H)** of wild-type mice and knockout mice both (receiving bone marrow from wild-type mice). Data **(A–H)** are representative of three to four mice for each group. Error bars, mean ± SEM. *P* > 0.05 (ns), *P* ≤ 0.05 (*), *P* ≤ 0.01 (**), *P* ≤ 0.0001 (****).

We accidentally observed CD4^+^ T-cell deficiency in mice that were obtained from long-term homozygous breeding of *lc3b* knockout mice. The population of CD4^+^ T cells was significantly decreased in the thymus (**Figures 1A, B** **)** and spleen (**Figures 1C, D** **)** of these mice. However, this phenotype was not observed in the original mice, which were not products of long-term homozygous breeding (**Figures S1A–D**). This indicated that the impairment of CD4^+^ T cells in these mice was independent of the original gene knockout. The development of CD4^+^ T cells involves two critical processes: differentiation from progenitor cells in the bone marrow and TCR- and MHC II-mediated T-cell maturation in the thymus. To determine the stage at which CD4^+^ T-cell development was impaired, we performed bone marrow transfer experiments. Wild-type mice that received the bone marrow of knockout mice had a CD4^+^ T-cell population similar to those receiving the bone marrow of wild-type mice (**Figures 1E, F** **)**. However, receiving the bone marrow of wild-type mice failed to rescue the CD4^+^ T-cell defect in knockout mice (**Figures 1G, H** **)**. These data suggest that CD4^+^ T-cell deficiency in knockout mice likely occurs in the thymus.

**Figure 2:**
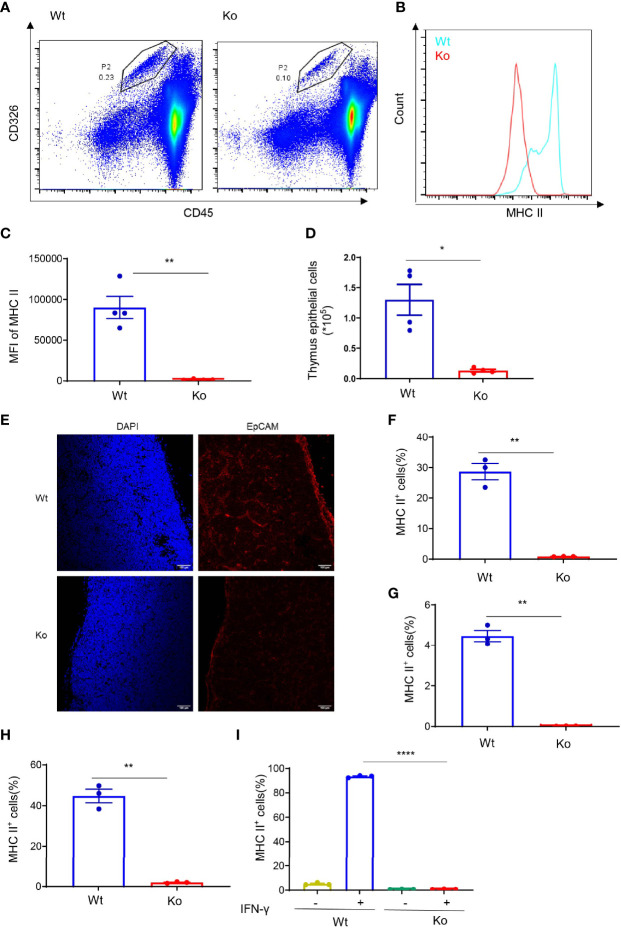
Decreased MHC II expression in the specific mouse strain. **(A)** Representative images of flow cytometry of thymus epithelial cells in wild-type and knockout mice. **(B, C)** Expression of MHC II in thymus epithelial cells of wild-type and knockout mice. **(B)** Overlaid histogram of flow cytometry. **(C)** MFI of MHC II. **(D)** Number of thymus epithelial cells in wild-type and knockout mice. **(E)** Immunofluorescence staining of CD326 in the frozen section of thymus tissue from wild-type and knockout mice. **(F–H)** Expression of MHC II in bone marrow **(F)**, thymus **(G)**, and spleen **(H)** of wild-type and knockout mice. **(I)** Expression of MHC II in bone marrow-derived macrophages of wild-type and knockout mice treated with 20 ng/ml IFN-γ for 24 h. Data **(C, D, F–I)** are representative of three to four mice for each group. Error bars, mean ± SEM. *P* ≤ 0.05 (*), *P* ≤ 0.01 (**), *P* ≤ 0.0001 (****).

After common lymphocyte progenitors migrate from the bone marrow to thymus, positive selection and negative selection are required for normal T-cell development. MHC II plays an important role in T-cell selection, especially in CD4^+^ T cells. Interestingly, we found that MHC II expression in the thymus epithelial cells of knockout mice was significantly lower than that in wild-type mice (**Figures 2A–C**). In addition, the number of thymus epithelial cells was significantly lower than that in wild-type mice (**Figure 2D**). We also performed immunofluorescence staining of CD326 in the frozen section of the thymus tissue (**Figure 2E**). The results were consistent with flow cytometry which indicated that the absolute number of thymic epithelial cells was decreased in the knockout mice. Moreover, overexpressing CD4^+^ TCR by crossing with OT-II mice failed to rescue their phenotype (**Figures S2A, B** **)**, which revealed that the extraordinary CD4^+^ T-cell population was not dependent on specific TCR rearrangement. We also checked MHC II expression in the bone marrow, thymus, and spleen (**Figures 2F–H**), and the data demonstrated that the decline of MHC II expression in these mice was generally observed in different immune cell types. Macrophages express a low level of MHC II in the normal state but display high MHC II expression upon IFN-γ treatment. Interestingly, IFN-γ failed to induce the expression of MHC II in macrophages from knockout mice (**Figure 2I**). These data suggest that CD4^+^ T-cell deficiency in these mice resulted from the loss of MHC II expression.

**Figure 3:**
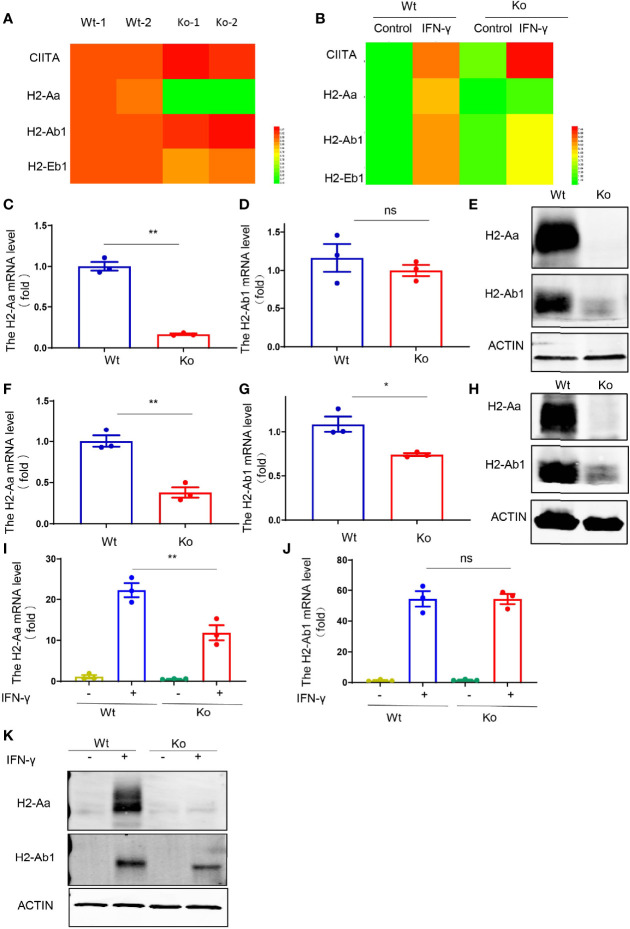
Reduced H2-Aa protein expression in specific mouse strain. **(A, B)** Heatmap of MHC II-associated gene expression obtained using RNA sequencing of bone marrow dendritic cells **(A)** and IFN-γ treated bone marrow-derived macrophages **(B)** in wild-type and knockout mice. **(C–E)** Expression levels of mRNA and protein **(E)** of H2-Aa **(C)** and H2-Ab1 **(D)** in bone marrow dendritic cells of wild-type and knockout mice. **(F–H)** Expression levels of mRNA and protein **(H)** of H2-Aa **(F)** and H2-Ab1 **(G)** in spleen B cells of wild-type and knockout mice. **(I–K)** Expression levels of mRNA and protein **(K)** of H2-Aa **(I)** and H2-Ab1 **(J)** in bone marrow-derived macrophages treated with IFN-γ for 24 h in wild-type and knockout mice. Data **(C, D, F, G, I, J)** were representative of three independent experiments. Error bars, mean ± SEM. *P* > 0.05 (ns), *P* ≤ 0.05 (*), *P* ≤ 0.01 (**).

To further investigate the cause of MHC II defects in this mouse strain, we performed RNA-seq with dendritic cells and IFN-γ-treated macrophages from knockout and wild-type mice. Only the expression of *H2-Aa* was significantly reduced in the knockout mice, while H2-Ab1 displayed a similar expression level (**Figures 3A, B** **)**. We confirmed this phenomenon in isolated dendritic cells, B cells, and IFN-γ-treated macrophages. The results showed that the H2-Aa protein was almost completely absent in knockout mice (**Figures 3E, H, K** **)**. However, the mRNA was still notably retained, although it was significantly decreased compared with the wild-type controls (**Figures 3C, F, I** **)**. In contrast, the H2-Ab1 mRNA remained the same in knockout mice (**Figures 3D, G, J** **)**, but the protein was decreased significantly (**Figures 3E, H, K** **)**. However, IFN-γ could induce the expression of H2-Ab1, not H2-Aa, in macrophages of knockout mice. These data suggest that the knockout mice lacked H2-Aa, mainly at the post-transcriptional level. As a subunit of MHC II, the loss of H2-Aa could result in MHC II deficiency and subsequently lead to an abnormal CD4^+^ T-cell population.

**Figure 4:**
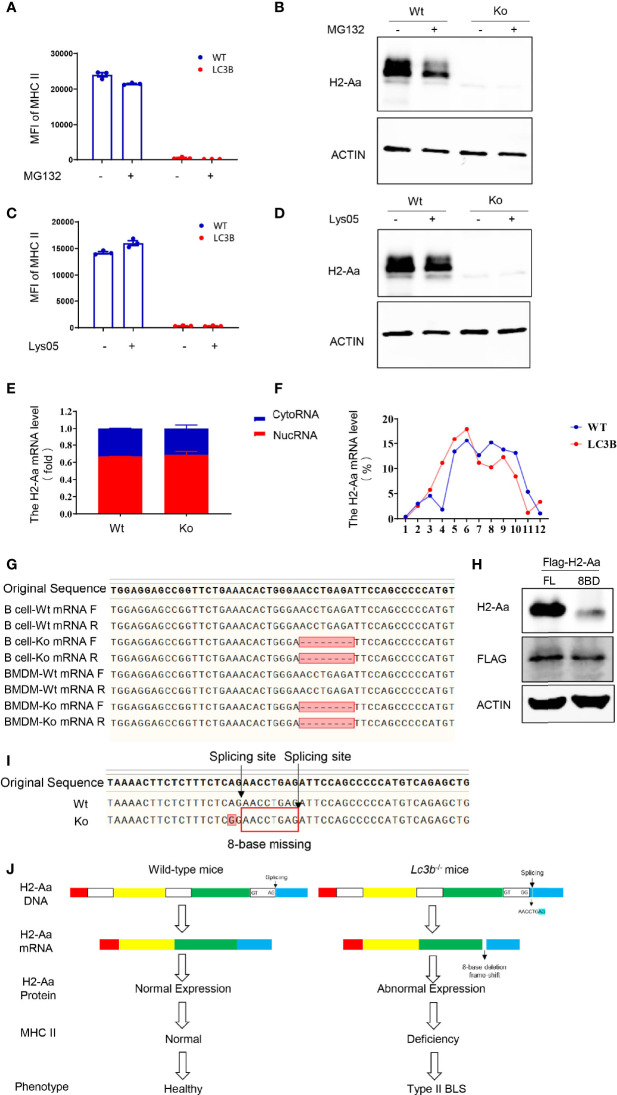
A point mutation on H2-Aa results in pre-mRNA false splicing and protein frameshift in this specific mouse strain. **(A, B)** MHC II **(A)** and H2-Aa **(B)** expression of B cells treated with MG132 in wild-type and knockout mice. **(C, D)** MHC II **(C)** and H2-Aa **(D)** expression of B cells treated with Lys05 in wild-type and knockout mice. **(E)** Respective H2-Aa expression in the nucleus and cytoplasm in B cells of wild-type and knockout mice. **(F)** Analysis of ribosome profiles of B cells in wild-type mice and knockout mice and H2-Aa expression was detected at each stage of ribosome using quantitation real-time PCR. **(G)** Forward and reverse sequences of B cells and bone marrow-derived macrophage cDNA in wild-type and knockout mice were obtained and aligned. **(H)** 293T cells were transfected with full-length or eight-base deletion Flag-H2-Aa plasmid and the expressions of Flag and H2-Aa were detected by Western blot. **(I)** DNA of bone marrow-derived macrophages in wild-type and knockout mice was isolated and sequences were aligned. A point mutation was observed and it could induce wrong pre-mRNA splicing and deletion of eight bases in mRNA. **(J)** The molecular biology model of type II BLS phenotype in *lc3b^−/−^* mice. A mutation from A to G caused the splicing recognition to malfunction until the next AG position Therefore, in *lc3b^−/−^* mice, wrong pre-mRNA splicing, deletion of eight bases in mRNA, and frameshift of H2-Aa protein eventually resulted in the loss of H2-Aa protein and subsequent immunodeficiency. Data **(A, C)** are replicated in three independent experiments. Error bars, mean ± SEM.

Furthermore, we explored the mechanisms of the loss of H2-Aa protein expression. We treated the B cells of knockout mice with MG132, a proteasome inhibitor, and Lys05, a lysosome inhibitor, and both failed to rescue MHC II deficiency and H2-Aa deletion. Moreover, MG132 alone induced a slight reduction in H2-Aa expression (**Figures 4A–D**). This indicated that the loss of H2-Aa was not a result of increased protein degradation. Translocation from the nucleus to the cytoplasm and movement of the ribosome are required for mRNA translation to proteins. To determine whether the nuclear plasma transport process of H2-Aa was normal, we performed nuclear mass separation, which was successful in detecting the mRNA levels of metastasis-associated lung adenocarcinoma transcript 1 (malat1) and the levels of Histone H3 and Calregulin proteins (**Figures S3A, B** **)**. We detected the H2-Aa mRNA levels in the nucleus and cytoplasm, and the data showed that there was no difference between the knockout and wild-type mice (**Figure 4E**), which indicated that the mRNA of H2-Aa could be smoothly transported to the cytoplasm after transcription in the nucleus. In the following study, we analyzed the ribosome profile of B cells in knockout and wild-type mice and isolated ribosomes at different stages to detect the *H2-Aa* mRNA level. The data demonstrated that B cells in knockout mice had a ribosome profile similar to that of wild-type mice (**Figure S3C**). *H2-Aa* mRNA was present at each stage of the ribosome, which was the same as that in the wild-type mice (**Figure 4F**). This indicated that the movement of *H2-Aa* mRNA on ribosomes was normal. Therefore, the defects did not arise during transport from the nucleus to the cytoplasm or during movement of ribosomes, nor did the defects occur due to protein degradation. Hence, we hypothesized that the H2-Aa protein loss might result from false mRNA. We then performed sequencing analysis and surprisingly found a deletion of eight bases of *H2-Aa* mRNA (**Figure 4G**). We treated 293T cells with full-length or eight-base deletion Flag-H2-Aa plasmid, and then detected the H2-Aa or Flag expression by Western blotting. Although there was no difference in Flag expression, H2-Aa was not observed in the eight-base deletion group (**Figure 4H**). These results indicated that eight-base deletion in the mRNA could lead to frameshift of the protein. To further understand the presence of false mRNA of *H2-Aa*, we sequenced the DNA of knockout mice and found a spontaneous point mutation in *H2-Aa*. The key to recognition of pre-RNA splicing is to determine the splicing site, which complies with the GT-AG rule. The mutation from A to G caused the splicing recognition to malfunction until the next AG position (**Figure 4I**). Therefore, in knockout mice, wrong pre-mRNA splicing, deletion of eight bases in mRNA, and frameshift of H2-Aa protein eventually resulted in the loss of this protein and subsequent immunodeficiency. Based on all these results, we delineated a model to clarify the consequences of the mutation leading to low expression of MHC class II and type II BLS phenotype (**Figure 4J**).

## Discussion

In this study, we observed impaired MHC II synthesis and CD4^+^ T-cell development when *lc3b* knockout mice underwent long-term homozygous breeding. Although autophagy was shown to play an essential role in T-cell proliferation, survival, and activation (11), knockout of autophagy-related protein ATG5 displayed no influence on T-cell development in the thymus (12). In addition, autophagy contributes significantly to MHC II antigen presentation, but not to MHC II expression (13–16). However, in the process of long-term homozygous breeding, *lc3b^−/−^* mice exhibited an *H2-Aa* spontaneous point mutation and consequent MHC II loss and CD4^+^ T-cell deficiency. However, why this mutation occurs in these knockout mice and whether the mutated gene and mutation site are specific remain to be clarified. A previous study revealed a relationship between autophagy and DNA damage. Autophagy plays an important role in DNA damage response (DRR) and autophagy is induced during DDR. Autophagy is crucial to genome stability and inhibiting autophagy may induce genomic instability and DNA damage response (17, 18). Thus, it is plausible that the mutation observed in *lc3b^−/−^* mice might have resulted from genomic instability; however, this requires further investigation.

We observe that CD4^+^ T-cell deficiency in this knockout mouse strain resulted from a reduction in MHC II expression. In addition, there were fewer thymus epithelial cells in the knockout mice than in the wild-type controls. However, we could not conclude that the reduced number of TECs resulted from the lack of MHC class II. Saldana et al. found that sonic hedgehog can regulate the differentiation of thymus epithelial cells. Conditional deletion of sonic hedgehog from TECs in the adult thymus results in alterations in TEC differentiation and consequent changes in T-cell development. Although TEC numbers are reduced, the cell surface expression of MHC II on medullary TECs was increased (19). This suggests that there is no direct relationship between thymus epithelial cell differentiation and MHC II expression. Therefore, in this study, the reduction of TEC number in this knockout mouse strain may be at least partially independent of MHC II deficiency induced by the *H2-Aa* point mutation. Theoretically, both MHC II deficiency and loss of TEC can impair CD4^+^ T-cell development. In the current study, we focused on the mechanistic study of MHC II deficiency, but paid little attention to the mechanisms of TEC loss. Liang et al. demonstrated that mTOR is essential for TEC development and maturation by regulating proliferation and Wnt signaling activity through autophagy (20). Thus, LC3B or autophagy could contribute to the reduction of thymus epithelial cells.

In this study, H2-Aa loss led to MHC II deficiency in *lc3b^−/−^* mice. In B cells and BMDC, the mRNA levels of H2-Ab1 were slightly but not significantly decreased in the knockout mice; however, the protein levels were significantly decreased compared with the wild-type controls. Interestingly, IFN-γ could induce the expression of H2-Ab1, for both mRNA and protein, in BMDM derived from *lc3b^−/−^* mice. Besides, there were no mutations in H2-Ab1 (data not shown). It is not clear why the H2-Ab1 protein was decreased. One plausible explanation is that H2-Aa deficiency might lead to the function loss of H2-Ab1, and thus, the translated H2-Ab1 protein was degraded.

In this study, MG132 and Lys05 failed to rescue MHC II and H2-Aa deficiencies. This suggested that protein degradation is not the cause of H2-Aa reduction in knockout mice. In addition, we found that MG132 alone reduced the expression of H2-Aa. Tewari et al. demonstrated a cytosolic pathway for MHC class II-restricted antigen processing, which was dependent on the proteasome and TAP (21). Bhat et al. further pointed out that Sug1, a 19S proteasome ATPase, played a critical role in the transcription of MHC class II, and the absence of the ATPase-binding domain increased the half-life of CIITA, but blocked MHC class II surface expression (22). These studies demonstrated the intimate connection between MHC II expression and the proteasome. These results are consistent with those of previous studies and provide a useful complement. The molecular mechanisms underlying the proteasome-dependent regulation of H2-Aa expression need to be clarified further.

MHC II is a crucial immune regulator, whose abnormal expression can lead to immune deficiency. Type II BLS is a rare, autosomal recessive immunodeficiency disease. Cells from typical type II BLS patients lack constitutive and inducible expression of all MHC II genes, similar to the phenotype observed in *lc3b^−/−^* mice used in this study. Therefore, these patients exhibited severely impaired T-cell activation and significantly reduced CD4^+^ cell number. The MHC II genes have two loci in mice: I-A and I-E. Mice of the H2b haplotype lack the expression of MHC II Ea molecule due to a loss-of-function deletion of about 600 bp in the promoter and first exon of the Ea gene, which results in the deficiency of I-E (23). However, our RNA-seq results showed that the mice used in this study exhibited a rather lower H2-Ea expression compared with H2-Aa (data not shown), indicating that the MHC II expression in these cells and mice was principally based on H2-IA. Therefore, this study focused on H2-IA, but not on H2-IE. To date, due to the lack of I-A, each type II BLS patient has a specific defect in a transcription factor that is necessary for MHC class II expression. Wiszniewski et al. described a family with an L469 mutation in *CIITA* that presented as an attenuated case, accompanied by residual MHC II expression in their report (24). Apart from *CIITA*, genes such as *RFXANK*, *RFX5*, and *RFXAP* were also affected in patients with type II BLS. The proteins encoded by these genes formed a complex that regulated the expression of MHC II. Some atypical cases still exist where the genetic defect is undefined. In this study, we observed a new paradigm of type II BLS in *lc3b^−/−^* mice, demonstrating a mechanism that is different from those reported in previous studies. Although these findings were observed in mice, this study provides a new potential mechanism of type II BLS.

Pre-mRNA splicing is required for the maturation of mRNA. The key to recognition of pre-RNA splicing is to determine the splicing site, which complies with the GT-AG rule. The composition of the 5′ splice site, or donor splice site, includes a nearly invariant “GT” dinucleotide sequence along with less conserved residues downstream. In rare cases, a “GC” dinucleotide also can be documented to serve as the 5′ splice site, while an “AG” at the 3′ end is required in the 3′ splice site or in the acceptor splice site (25). In this study, a point mutation from A to G at the 3′ end of H2-Aa caused the splicing recognition to malfunction until the next AG position, which induced the deletion of eight bases in mRNA and the eventual loss of this protein. The eight-base deletion appeared in the origination of exon 5 and led to a change in the amino acid sequences of this exon. We noted that the transmembrane domain and intracellular domain of H2-Aa were both located in this exon, and abnormal transmembrane domain could greatly influence the expression of the H2-Aa protein, likely resulting in the almost complete absence of H2-Aa in knockout mice.

In summary, we observed a spontaneous *H2-Aa* point mutation in mice obtained from the homozygous breeding of *lc3b^−/−^* mice. This mutation induced the errant splicing of pre-mRNA, eight-base deletion of mRNA, frameshift of protein, and consequent deficiency of MHC II. However, whether LC3B or autophagy specifically contributed to this point mutation requires further investigation.

## Materials and Methods

### Mice

*Lc3b^−/−^* and OT-II mice (C57BL/6) were originally obtained from the Jackson Laboratory and housed at Zhejiang University Laboratory Animal Center. *Lc3b^−/−^* mice were bred homozygously for approximately 10 generations. More detailed information on the animal experiments is provided below. All experimental protocols were approved by the Ethical Committee for Animal Studies at Zhejiang University in 2021. The animal ethics approval number was ZJU20210302 and the period of validity was 4 years.

### Antibodies and Primers

Antibodies used for flow cytometry were purchased from eBioscience (San Diego, U.S.A.): CD4 (GK1.5), CD8 (53-6.7), CD45 (30-F11), CD326 (G8.8), MHC II (M5/114.15.2), and B220 (RA3-6B2). The antibodies used for Western blot were as follows: Calregulin and Histone H3 were obtained from Santa Cruz Biotechnology (Dallas, U.S.A.), Flag (D190828) was purchased from Sangon Biotech (Shanghai, China), and H2-Aa (A18325) and H2-Ab1 (A18658) were custom-made from ABclonal Technology (Wuhan, China). The antibody CD326 for IF was obtained from Santa Cruz Biotechnology. The primers used for quantitative real-time PCR were as follows: *Actb* forward: 5′-AGAGGGAAATCGTGCGTGAC-3′, reverse: 5′-CAATAGTGATGACCTGGCCGT-3′; *H2-Aa* forward: 5′-CTGACCACCATGCTCAGCCTCT-3′, reverse: 5′-TACTGGCCAATGTCTCCAGGAG-3′; *H2-Ab1* forward: 5′-ACCCAGCCAAGATCAAAGTGC-3′, reverse: 5′-TGCTCCACGTGACAGGTGTAGA-3′; *Malat1* forward: 5′-CACTTGTGGGGAGACCTTGT-3′, reverse: 5′-GTTACCAGCCCAAACCTCAA-3′; *U6* forward: 5′-CGCTTCGGCAGCACATATACTAAAATTGGAAC-3′, reverse: 5′-GCTTCACGAATTTGCGTGTCATCCTTGC-3′. The primers used for sequencing were as follows: DNA sequencing: 5′-GTGTGTATGAGCTCTGTCATCTTCTGCACTT-3′, reverse: 5′-TCATAAAGGCCCTGGGTGTCT-3′; cDNA sequencing: forward: 5′-CTGACCACCATGCTCAGCCTCT-3′, reverse: 5′-TCATAAAGGCCCTGGGTGTCT-3′.

### Preparation of B Cells, Dendritic Cells, and Bone Marrow-Derived Macrophages

B cells were isolated from mouse spleens using magnetic bead separation. Dendritic cells and macrophages were derived through bone marrow-induced differentiation. GM-CSF (20 ng/ml) was used for dendritic cell differentiation, while M-CSF (10 ng/ml) was used for macrophage differentiation. In addition, macrophages were subjected to IFN-γ (20 ng/ml) treatment to induce MHC II expression.

### Bone Marrow Transfer

Bone marrow transfer (BMT) was performed by transplanting the total BMCs (5 × 10^6^) from 8-week-old male mice (donor) into lethally irradiated (X-ray radiation at a dose of 8 Gy) 8-week-old male mice (recipient) through the tail vein. Phenotype analysis was conducted 2 months after BMT.

### Nuclear and Cytoplasm Separation

The cell pellet was collected and suspended in 5× volume-cell-weight CE buffer (10 mM HEPES, pH 7.9, 1.5 mM MgCl2, 10 mM KCl, proteinase inhibitor cocktail, and fresh 0.075% NP-40). The cells were incubated on ice for 10 min and centrifuged at 1500rpm for 10 min at 4°C. The supernatant and pellet contained the cytoplasm and nuclei, respectively. The nuclei were with CE buffer without NP-40 to avoid cytoplasmic contamination. Moderate AG RNAex Pro Reagent was added for RNA extraction.

### Ribosome Profile

Before collection, purified B cells were incubated with 100 μg/ml cycloheximide (CHX) for 5 min at 37°C. The cells were then washed twice with PBS containing 100 μg/ml CHX, 1× protease inhibitor cocktail, and 100 U RNase inhibitor and pelleted using centrifugation. Cell pellets were lysed in 500 μl lysis buffer (200 mM KCl, 15 mM MgCl_2_, 20 mM HEPES pH 7.5, 0.5% Triton X-100, 100 μg/ml CHX, 100 U RNase inhibitor, 1× protease inhibitor cocktail, 1 mM DTT) and incubated for 15 min on ice and centrifuged at 14,000 rpm for 5 min at 4°C. The supernatants were layered over a 10%–50% sucrose gradient plus 100 μg/ml CHX, 1× protease inhibitor cocktail, and 1 mM DTT and centrifuged at 38,000 rpm for 2 h at 4°C using Beckmann Coulter SW41 Ti rotors. Gradients were fractionated using a BioComp Piston Gradient Fractionator with continuous A260 measurements. Twelve fractions were manually collected from each gradient.

### Plasmid Transfection

Plasmid transfection was performed with polyethylenimine, linear MW 25,000 (Polysciences) following the manufacturer’s protocol. Cells were seeded to each well of six-well plates for 24 h before transfection. The infection medium was replaced with fresh growth medium after being incubated with 293T cells for 36 h. Cells were collected for Western blot assay.

### RNA Isolation and Quantitative Real-Time PCR Analysis

Total RNA was extracted from the total cellular, nuclei, cytoplasmic, and ribosomal fractions using AG RNAex Pro Reagent (AG21102). RNA was reverse-transcribed using Evo M-MLV RT Premix for qPCR (AG11706), and cDNA was used for quantitative real-time PCR with SYBR Green Premix Pro Taq HS qPCR Kit (AG11701) on a StepOne Real-Time PCR System (Applied Biosystems, Foster City, CA, USA) to determine the expression levels of mouse *H2-Aa*, *Malat1*, and *U6.* The products used above were purchased from Accurate Biotechnology (Hunan) Co., Ltd. (Changsha, China) and all procedures were performed according to the manufacturer’s protocol. The data were normalized to *Actb* expression levels. The primers used are listed above.

### Western Blot Assay

Cells were prepared with RIPA lysis buffer (Beyotime, Shanghai, China, P0013B) in the presence of protease inhibitor cocktail (Roche Diagnostics GmbH, 04-693-116-001). Lysates were loaded to SDS–PAGE and immunoblotted with relevant antibodies using standard methods.

### Flow Cytometry

Cytoflex (Beckman Coulter) was used to identify cell surface markers. Cell sorting was performed by Moflo Astrios EQ (Beckman Coulter). All the results were analyzed by CytExpert or FlowJo X software. Fluorescence-conjugated antibodies are listed above.

### Immunofluorescence Staining

Thymus tissues were embedded in the optimal cutting temperature (OCT) and cut into 6 μm pieces at −20°C for immunofluorescence staining. The frozen sections were stained with anti-CD326 according to the manufacturer’s protocol. Fluorescent images were captured with an Olympic FV3000 laser scanning confocal microscope.

### Statistical Analyses

Statistical tests were performed using GraphPad Prism software (version 9.0; San Diego, CA, USA). Data were assessed using a parametric statistical test (*t*-test for differences between two groups, one-way ANOVA for those between multiple groups) and presented as mean ± SEM. Differences were considered significant if *P* ≤ 0.05 (*), *P* ≤ 0.01 (**), *P* ≤ 0.001 (***), and *P* ≤ 0.0001 (****).

## Data Availability Statement

The original contributions presented in the study are included in the article/**Supplementary Material**. Further inquiries can be directed to the corresponding authors.

## Ethics Statement

The animal study was reviewed and approved by the Ethical Committee for Animal Studies at Zhejiang University. Written informed consent was obtained from the owners for the participation of their animals in this study.

## Author Contributions

H-HS, Z-HC, and LW designed and supervised the study. YZ, JX, H-XC, MZ, L-NZ, Y-FW, W-JL, XF, FL, and CZ performed the experiments. WL and S-MY supervised the experiments. YZ and JX prepared the figures. YZ, H-HS, Z-HC, and LW drafted the manuscript. All authors contributed to the article and approved the submitted version.

## Funding

This work was supported by the Major Project (82090012 to H-HS), General Projects (31970826 to Z-HC), and Key Project (81930003 to H-HS) from the National Natural Science Foundation of China, and the Major Project from the Natural Science Foundation of Zhejiang Province (LD21H010001 to Z-HC).

## Conflict of Interest

JX was employed by Qihan Bio Inc.

The remaining authors declare that the research was conducted in the absence of any commercial or financial relationships that could be construed as a potential conflict of interest.

## Publisher’s Note

All claims expressed in this article are solely those of the authors and do not necessarily represent those of their affiliated organizations, or those of the publisher, the editors and the reviewers. Any product that may be evaluated in this article, or claim that may be made by its manufacturer, is not guaranteed or endorsed by the publisher.

## Supplementary Material

Supplementary Figure 1 The CD4^+^ T cell deficiency of the mice is independent of the original gene knockout. **(A, C)** Representative images of flow cytometry of thymus **(A)** and spleen**(C)** in wild-type and original knockout mice. **(B, D)** Proportion of CD4^+^ T cells in thymus **(B)** and spleen **(D)** in wild-type and original knockout mice. Data **(B, D)** are representative of 3–4 mice for each group. Error bars, mean ± SEM.

Supplementary Figure 2 CD4^+^ TCR overexpression fails to rescue CD4^+^ T cell deficiency in the specific mouse strain. **(A, B)** Knockout mice were crossbred with OT-II mice to get four genotypes: Wt mice, Ko mice, OT-II mice, and OT-II- Ko mice. T cell population detecting using flow cytometry of thymus **(A)** and spleen**(B)** in these four genotypes.

Supplementary Figure 3 Quality control of cytoplasmic separation and ribosome profile. **(A)** The respective nuclear and cytoplasmic *Malat1* expression of in B cells of wild-type and knockout mice. **(B)** Calregulin and Histone H3 expression in nuclear and cytoplasm extracts for quality control of cytoplasmic separation. **(C)** Ribosome profile of B cells in wild-type and knockout mice. **(D)** *U6* expression in each stage of ribosome for quality control of ribosome profile.
