# Supplementary figures and images for: A Spontaneous H2-Aa Point Mutation Impairs MHC II Synthesis and CD4+ T-Cell Development in Mice
Source: Front Immunol. 2022 Mar 4;13:810824. doi: 10.3389/fimmu.2022.810824 (PMC8931304; doi:10.3389/fimmu.2022.810824)

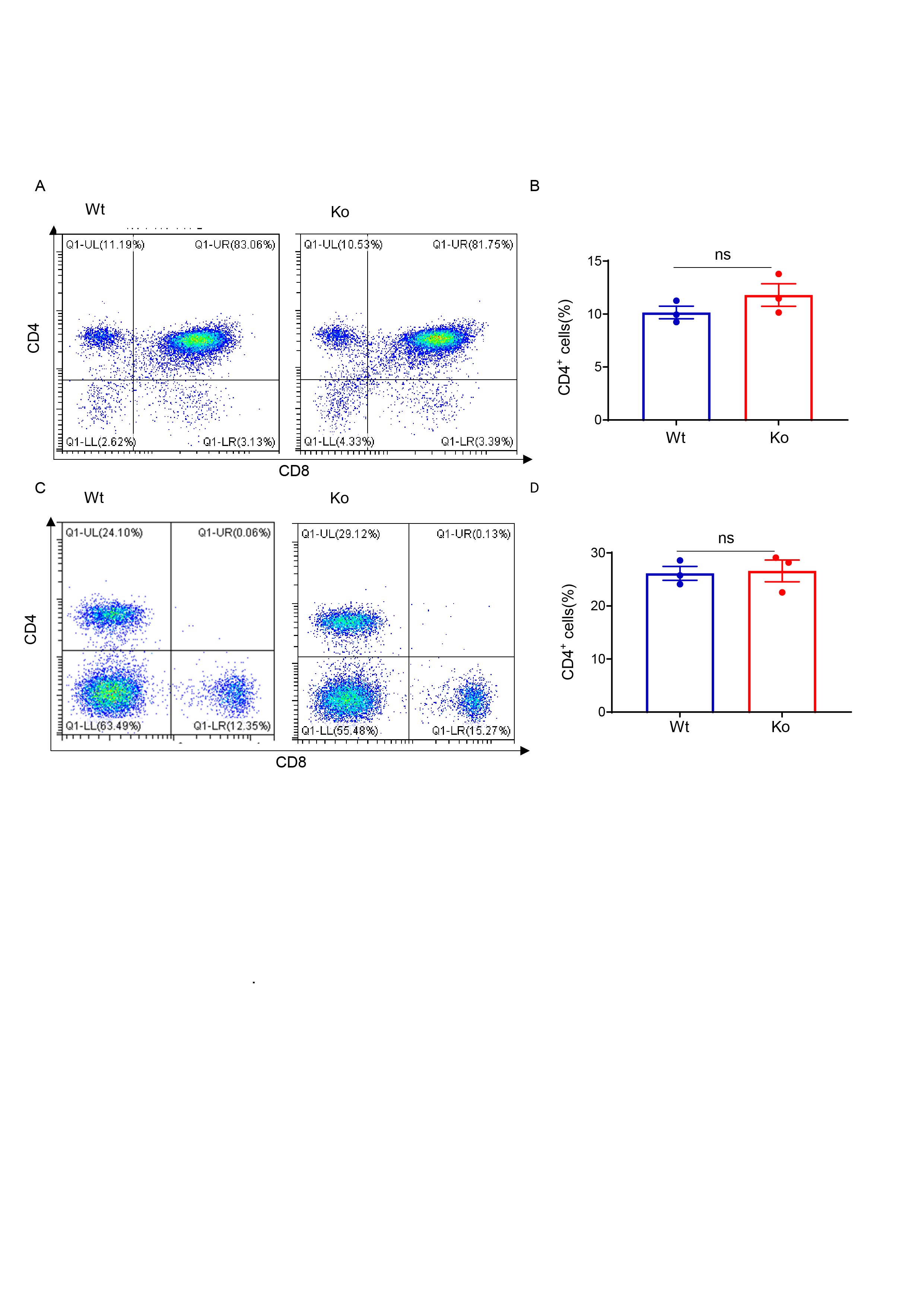

Supplement: Supplementary Figure 1 — The CD4+ T cell deficiency of the mice is independent of the original gene knockout. (A, C) Representative images of flow cytometry of thymus (A) and spleen(C) in wild-type and original knockout mice. (B, D) Proportion of CD4+ T cells in thymus (B) and spleen (D) in wild-type and original knockout mice. Data (B, D) are representative of 3–4 mice for each group. Error bars, mean ± SEM. [file Image_1.jpeg]

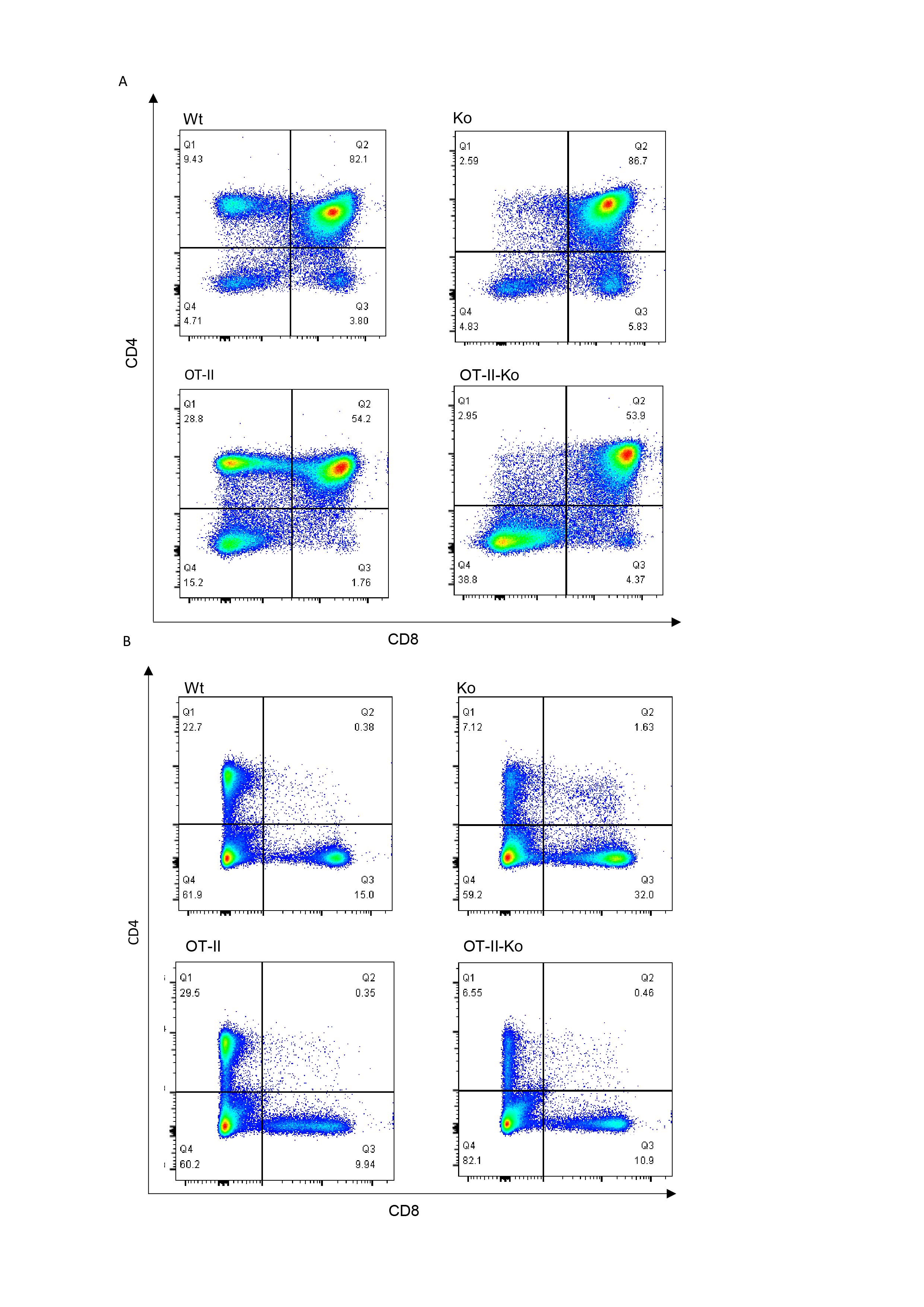

Supplement: Supplementary Figure 2 — CD4+ TCR overexpression fails to rescue CD4+ T cell deficiency in the specific mouse strain. (A, B) Knockout mice were crossbred with OT-II mice to get four genotypes: Wt mice, Ko mice, OT-II mice, and OT-II- Ko mice. T cell population detecting using flow cytometry of thymus (A) and spleen(B) in these four genotypes. [file Image_2.jpeg]

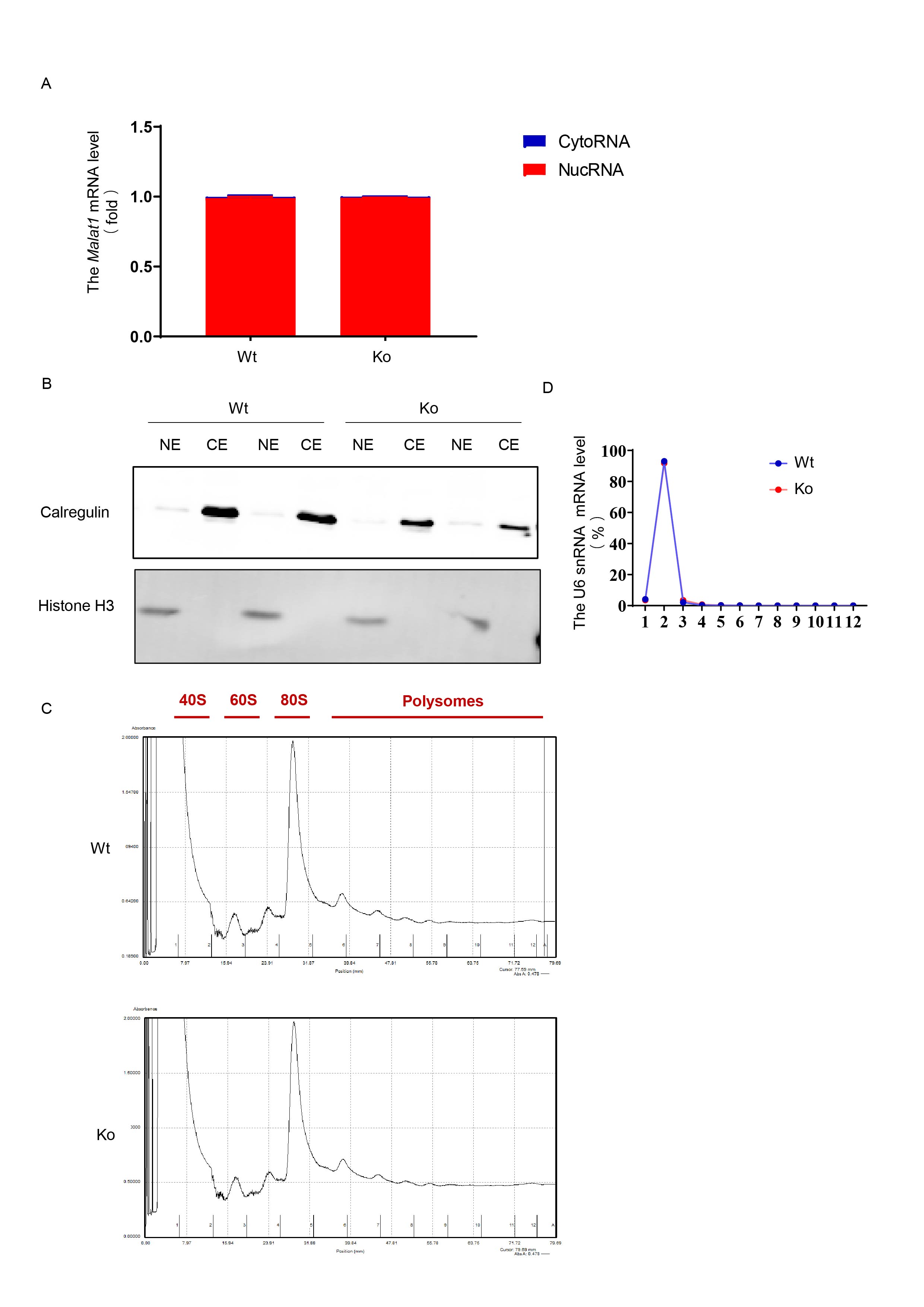

Supplement: Supplementary Figure 3 — Quality control of cytoplasmic separation and ribosome profile. (A) The respective nuclear and cytoplasmic Malat1 expression of in B cells of wild-type and knockout mice. (B) Calregulin and Histone H3 expression in nuclear and cytoplasm extracts for quality control of cytoplasmic separation. (C) Ribosome profile of B cells in wild-type and knockout mice. (D) U6 expression in each stage of ribosome for quality control of ribosome profile. [file Image_3.jpeg]
